# Rapid Deployment of Telemedicine in HIV Care: Mixed Methods Study of Providers’ Attitudes and Perceptions

**DOI:** 10.2196/75933

**Published:** 2026-04-27

**Authors:** Shannon Galvin, Artur Queiroz, Juan Rivera, Mia Calamari, Katherine Wright, Lisa R Hirschhorn, Laura Rusie, Patrick Janulis, Rebecca Kumar, Frank Palella, Mary Clare Masters, Jacqueline Bannon, Susheel Reddy, Claudia Hawkins

**Affiliations:** 1 Division of Infetious Disease, Northwestern University, 645 N Michigan Avenue, Chicago, IL, 60611, United States, +1 312-695-2373; 2 Department of Nursing, Florida State University, Tallahassee, FL, United States; 3 Howard Brown Health Center, Chicago, IL, United States; 4 Department of Medical Social Sciences, Northwestern University, 645 N Michigan AvenueChicago, IL, 60611, United States; 5 Georgetown University, Washington, DC, United States

**Keywords:** telemedicine, HIV, provider attitudes, telehealth, mixed methods

## Abstract

**Background:**

The COVID-19 pandemic prompted an abrupt transition to telemedicine for HIV care, necessitating the exploration of provider attitudes and experiences to understand its ongoing viability and impact.

**Objective:**

This study aimed to assess providers’ attitudes toward and experiences with telemedicine at HIV clinics in the Chicago area during the COVID-19 pandemic at 2 time points and at 2 clinical sites.

**Methods:**

We conducted a convergent mixed methods study to evaluate and explore providers’ attitudes toward and experiences with telemedicine at HIV clinics in the Chicago area during the COVID-19 pandemic, applying the RE-AIM (reach, effectiveness, adoption, implementation, and maintenance) framework and the updated Consolidated Framework for Implementation Research. This study assessed HIV providers via surveys at 2 time points, capturing responses on the acceptability, appropriateness, feasibility, and maintenance of telemedicine. Semistructured key informant interviews were conducted on a random selection of 10 participants to explore perceived experiences with telemedicine.

**Results:**

Among emailed providers, 43 of 83 (51.8%) and 27 of 82 (32.9%) responded to survey 1 and survey 2, respectively. The first survey recorded telemedicine usage at 75%, which decreased to 58% by the second survey. Overall, the majority of respondents agreed with statements that assessed telemedicine as appropriate, acceptable, and feasible. There were overall few statistical differences in responses between sites, although more providers at the community site indicated at least some potential value in telemedicine when compared to providers at the university hospital (100% agree or strongly agree vs 82.3%; *P*=.04) in survey 1. In survey 1, providers with more than 10 years of experience were less likely to report telemedicine helped them see more patients (31.6% agree or strongly agree vs 70.6% for providers with ≤10 y of experience; *P*=.008). Fewer experienced providers felt they could talk about private issues with their patients during telemedicine (57.9% of more experienced vs 88.3% of less experienced providers; *P*=.03). Key informant interviews provided qualitative insights into telemedicine integration, revealing mixed sentiments; providers appreciated the flexibility and accessibility that telemedicine offered but preferred in-person visits for their thoroughness, especially for physical examinations and laboratory tests. Despite initial barriers such as technical challenges and patient preferences for in-person visits, telemedicine was deemed feasible for maintaining communication and care quality. Providers highlighted the need for better technological support and ongoing training to optimize telemedicine usage.

**Conclusions:**

Our study underscores telemedicine as a sustainable adjunct to traditional HIV care, emphasizing the importance of addressing technological and training barriers to enhance its efficacy.

## Introduction

The COVID-19 pandemic created abrupt and large-scale changes in health care delivery. One notable effect was the rapid increase in the use of telemedicine. Telemedicine is a type of telehealth in which telecommunications technologies support the delivery of medical treatment–related services [1]. A telehealth visit or televisit can be defined as a singular 2-way, synchronous client-provider interaction via telephone or video [2]. Telemedicine use increased from less than 1% of visits prior to the COVID-19 pandemic to as high as 80% in March to April 2020 [3]. While peak use was reported during the pandemic in the spring of 2020, telemedicine use remains high. Recent data show that, overall, 39% to 43% of US adults reported having a televisit in the past year [4,5]. Despite its widespread adoption, the future of telemedicine in the United States is unclear, in part, due to uncertainty in regulations and reimbursement.

HIV care reflected the national trend with a rapid increase in the adoption of telemedicine beginning in 2020. More than 99% of Ryan White providers offered telemedicine after the pandemic, compared to 22% before the pandemic [6]. In the US Department of Veterans Affairs system in 2023, HIV was one of the conditions with the highest proportion of care visits via telemedicine, with 8.8% of visits [7]. While televisits have been shown to be useful in HIV prevention, including improving HIV self-testing [8] and pre-exposure prophylaxis uptake [8-10], the use of telemedicine for HIV care is still evolving. Studies evaluating telemedicine in HIV care before the pandemic showed no difference in virologic outcomes and demonstrated effectiveness in mental health care delivery for persons living with HIV in select settings [11]. Studies conducted after the COVID-19 pandemic have started to document the use of telemedicine in HIV care, showing equivalent or better virologic outcomes when compared to in-person care [12]. The uptake, acceptability, and effectiveness of the rapid, widespread rollout of telemedicine for HIV care during the COVID-19 pandemic and beyond are still not completely understood. Potential benefits include increased patient access, improved retention, greater patient convenience, and a reduction in exposure to COVID-19 in health care settings [13,14]. Risks include a potential decrease in quality of care, concerns about confidentiality of data or personal information, especially related to HIV diagnosis, and inequities in care delivery based on patients’ access to technology [13,14].

HIV providers’ attitudes and experiences are critical to understanding the risks and benefits of telemedicine in HIV care and to providing important information on barriers and facilitators at the system, clinic, and patient levels. Providers’ attitudes are key factors in adopting telemedicine [15]. In a small study of HIV care organizations in South Carolina, providers frequently reported barriers to telemedicine, including insufficient technology support, lack of clear guidelines, lack of ability to assess laboratory data, and both client and provider unfamiliarity with the technology and the procedures [16]. In other studies of single HIV sites, overall provider acceptability of the use of telemedicine in general was high [17,18]. Whether telemedicine will be maintained as a viable strategy requires further information on HIV provider views and experiences.

We conducted a convergent mixed methods study to evaluate and explore providers’ attitudes toward and experiences with telemedicine at HIV clinics in the Chicago area during the COVID-19 pandemic, applying the RE-AIM (reach, effectiveness, adoption, implementation, and maintenance) framework and updated Consolidated Framework for Implementation Research frameworks [19,20]. We hypothesized that providers’ attitudes toward telemedicine would change over time and be associated with provider and clinic characteristics. Findings from this study could have important practical implications for the continued use of telemedicine in routine HIV clinical practice and future times of system stress.

## Methods

### Overview

This mixed methods study was nested within a larger study evaluating the processes, implementation outcomes, and effectiveness of an emergently implemented telemedicine program for HIV care during the COVID-19 pandemic, and its effectiveness for people living with HIV managed in an academic (Northwestern University [NU]) and a community outpatient clinic (Howard Brown Health Center [HB]) between March 13, 2020, and December 31, 2021 [21].

### Study Location and Telemedicine Implementation During the COVID-19 Pandemic

NU provides care for people living with HIV in an outpatient clinic based at Northwestern Memorial Hospital. The NU patient population is predominantly non-Hispanic Black and White men who have sex with men, most of whom are insured. HB provides care at 10 community-based sites to a racially diverse, gender-diverse, and culturally diverse population of people living with HIV, many (30%) of whom are underinsured or uninsured. People living with HIV managed at both sites are medically complex, with multiple comorbidities that further challenge adherence to care and treatment success.

In March 2020, NU and HB rapidly implemented telemedicine to minimize patient and provider exposure to COVID-19. Each clinic provided patients with the option to convert their scheduled in-person outpatient appointments to visits that could be conducted virtually via telephone or videoconference using Doximity (Doximity Inc.) (NU) or Health Insurance Portability and Accountability Act (HIPAA)–compliant health care version of Zoom (Zoom Communications Inc.) (HB). At both sites, if the provider determined that the patient required an in-person visit (before or during the televisit), they were scheduled with one of the on-site providers. If a patient required laboratory testing but had a televisit, they were instructed to come to the appropriate site for testing.

### Study Population and Procedures

All providers (nurse practitioners, physicians, and physician assistants) who provided direct care for persons with HIV at NU and HB during the study period were eligible. Providers were asked to complete 2 anonymous online surveys developed and administered using REDCap (Research Electronic Data Capture) between December 7, 2020, and March 12, 2021 (survey 1) and November 17, 2021, and January 10, 2022 (survey 2). Key informant interviews (KIIs) were then conducted among a subset of providers between August 2021 and November 2021. The mixed methods design was chosen, with a qualitative component (KIIs) giving context to the quantitative survey results.

The RE-AIM framework was selected to guide the quantitative evaluation of implementation outcomes, including reach, adoption, implementation, and maintenance [19]. The Consolidated Framework for Implementation Research (CFIR) was used to guide qualitative data collection and analysis, providing a structured approach to identifying multilevel determinants influencing implementation processes [20]. The use of both frameworks allowed RE-AIM to characterize *what* implementation outcomes were observed, while CFIR supported the interpretation of *why* these outcomes occurred by identifying contextual facilitators and barriers [22].

### Survey

A REDCap-generated email was sent to 83 providers (27 NU and 56 HB) that contained a link to the survey instrument. Survey recruitment continued until all providers had 3 opportunities to respond, at which point the recruitment was closed. The survey was adapted from prior validated instruments, including the System Usability Scale [23,24], Normalization Measure Development Questionnaire [25,26], and implementation outcomes modified for local context. The initial survey (survey 1) included questions on acceptability, appropriateness, feasibility, and maintenance of telemedicine during the COVID-19 pandemic, as well as additional questions on frequency and reasons for conducting telemedicine. The same survey (survey 2) was administered a second time in March 2021 to 82 providers (26 NU and 56 HB), with additional questions on how the number of telemedicine visits changed since the prior questionnaire, reasons for the change, if any, and preferences for future telemedicine visits. Survey questions in the domains of acceptability, appropriateness, feasibility, and maintenance were rated by participants using a 5-point Likert scale (ie, strongly agree, agree, neither agree nor disagree, disagree, and strongly disagree; Table S1 in Multimedia Appendix 1).

### Key Informant Interviews

KIIs were conducted with 10 participants. Providers were randomly selected from the eligible pool. After 10 participants, saturation of themes was deemed to have been met, consistent with literature assessing sample sizes for qualitative studies in homogeneous populations [27,28]. Study investigators reviewed and edited semistructured interview guides in discussion with other study team members. Study interviews were conducted over Zoom in a private room by the study coordinator, who was trained in qualitative techniques by investigators with this expertise. Study interviews lasted between 30 and 65 minutes and occurred over 2 months. Interview questions were open-ended and explored perceived experiences with telemedicine during the COVID-19 pandemic, features, and processes to integrate telemedicine, barriers and facilitators, and perceived differences in care with telemedicine.

All interviews were conducted in English, digitally recorded, and transcribed. Two authors (CH and SG) first read all transcripts independently and then jointly identified themes to guide the analysis. AQ conducted a lexical analysis with the software IRaMuTeQ-*Interface de R pour les Analyses Multidimensionnelles de Textes et de Questionnaires* (version 0.7), operating as an interface within R (version 4.4.2; R Foundation for Statistical Computing) [29-32].

For the textual analysis, the descending hierarchical classification method was defined, in which the texts are classified according to their respective words, and their set is divided by the frequency of the reduced forms. Thus, classes of text segments were obtained, which were called “preclass.” Subsequently, the organization of key expressions from the interviewed speeches and the identification of central ideas complementing the descending hierarchical classification findings were performed and allowed the delimitation of statements in definite classes [33]. AQ guided the deductive theming of data under each CFIR domain, and disagreements were resolved by discussion between the authors. CFIR’s 5 main domains and multiple constructs used were intended to capture barriers to and facilitators of implementation. The domains we explored included innovation characteristics (eg, relative advantage of telehealth vs in-person appointments, perceived adaptability of telehealth to meet the current local needs of patients during the pandemic), individual characteristics (eg, knowledge and beliefs about the intervention, and personal attributes such as motivation and capability to adapt to telehealth), inner setting (eg, capacity of the organization, the organizational implementation climate, and capacity for change), outer setting (eg, political and societal changes that can influence the implementation), and process (eg, characterized by plans to ease implementation and engagement of community stakeholders). Direct quotations are provided to illustrate the evidence from which the findings were derived, and themes are summarized.

### Statistical Analysis

Internal consistency and reliability of items within domains were assessed using raw Cronbach α. Alpha values indicated satisfactory internal consistency for the feasibility (α=0.846) and maintenance (α=0.899) domains. The α values for the appropriateness domain (α=0.672) were equivocal.

Responses were tabulated separately for each survey administration. Individual Likert items were summarized as ordinal categorical measures using frequency counts and percentages across the 5-point Likert scale (strongly agree to strongly disagree). Acknowledging the limited sample size and skewed distributions in many instances, exact Mantel-Haenszel chi-square statistics were used to identify differences in the distribution of responses by site (NU vs HB) and respondent tenure (≤10 y vs >10 y).

Survey administrators were blinded to the identity of respondents across the survey 1 and survey 2 administrations. Changes in perceptions for a given respondent were not tracked as a result, and direct inferential comparisons between survey 1 and survey 2 results were not applied. All analyses were completed using SAS (version 9.4). Data visualizations were prepared using Tableau Desktop (version 2021.3.1; Tableau Software).

### Ethical Considerations

The research was reviewed and approved by the Institutional Review Boards at NU (STU00213441) and HB (DEF 20‐26). Informed consent was obtained from all participants. Data for qualitative surveys were anonymous, and data for KIIs were deidentified after transcription. No compensation was provided to participants.

## Results

### Overview

A total of 43 of 83 (51.8%) and 27 of 82 (32.9%) emailed providers responded to survey 1 and survey 2, respectively. Of survey 1 respondents, 30.2% (13/43) had been providing HIV care for over 15 years, 16.3% (7/43) for 11 to 15 years, 41.9% (18/43) for 1 to 10 years, and 11.6% (5/43) for less than 1 year (Table 1). Distribution by tenure for survey 2 respondents was not markedly different, with 33.3% (9/27) providing care for over 15 years, 22.2% (6/27) for 11 to 15 years, 37% (10/27) for 1 to 10 years, and 7.4% (2/27) for less than 1 year.

**Table 1.** Respondent characteristics.

Characteristics	Survey 1 (n=43), n (%)	Survey 2 (n=27), n (%)
Tenure (years providing HIV care)		
<1	5 (11.6)	2 (7.4)
1‐2	8 (18.6)	4 (14.8)
3‐5	7 (16.3)	5 (18.5)
6‐10	3 (7)	1 (3.7)
11‐15	7 (16.3)	6 (22.2)
>15	13 (30.2)	9 (33.3)
Professional category		
Nurse practitioner	10 (23.3)	7 (25.9)
Physician	32 (74.4)	18 (66.7)
Physician assistant	1 (2.3)	2 (7.4)
Any telemedicine in survey periods		
No	7 (16.3)	3 (11.1)
Yes	36 (83.7)	24 (88.9)
Proportion of visits completed using telemedicine^a^		
<25%	19 (52.8)	16 (66.7)
25%‐75%	12 (33.3)	8 (33.3)
>75%	5 (13.9)	0 (0)

a Survey 1: n=36; and survey 2: n=24.

The proportion of health care encounters that were telemedicine declined between the 2 survey time points from 75% to 58%. In survey 1, a total of 19 of 36 (52.8%) reported conducting 25% to 75% of their HIV visits via telemedicine, with 12 of 36 (33.3%) conducting less than 25% and 5 of 36 (13.9%) conducting more than 75%, with one of those respondents conducting all visits via telemedicine. The proportion of providers conducting 25% to 75% telemedicine decreased to 33.3% (8/24) in survey 2, and none reported conducting more than 75%. Although telemedicine seemed to comprise a smaller proportion of a provider’s encounters in survey 2, more providers in survey 2 reported at least some use of telemedicine, with 7 of 43 (16.3%) reporting not completing any telemedicine visits in survey 1 versus 3 of 27 (11.1%) in survey 2. A minority of telemedicine visits were conducted by video in both time frames, with only 8 of 36 (22.2%) in survey 1 and 4 of 24 (16.7%) in survey 2 reporting more than 75% video use.

Those providers reporting no use of telemedicine cited having visits where procedures and same-day or urgent visits were needed as reasons. One provider reported never conducting telemedicine visits at all during the study, stating, “I do not feel comfortable doing telemedicine.”

The main reasons for not conducting a televisit included patients’ desire to be seen in person (survey 1: 28/36, 77.8%; and survey 2: 11/24, 45.8%), patients having conditions that required physical examination (survey 1: 29/36, 80.6%; and survey 2: 19/24, 79.2%), or the need for laboratory testing (survey 1: 26/36, 72.2%; and survey 2: 19/24, 79.2%).

### Telemedicine Outcomes Among Providers

**Figure 1.**
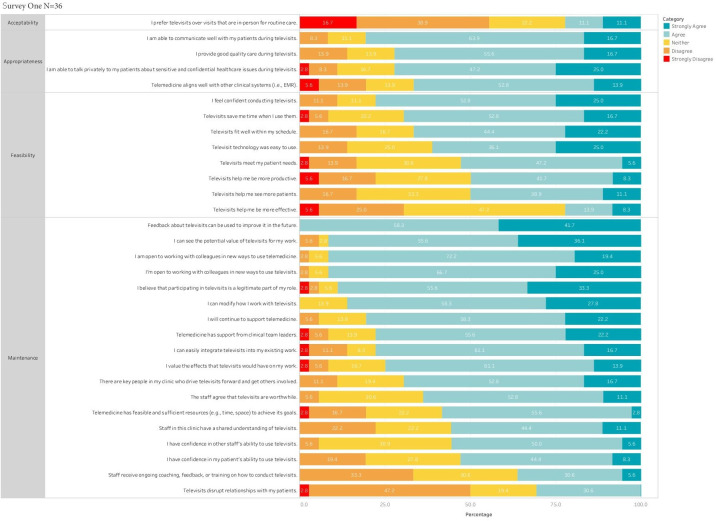
Survey 1.

Provider responses to survey 1 are shown in Figure 1. In survey 1, a total of 20 (55.6%) respondents disagreed with the statement, “I prefer telemedicine over in person visits,” indicating that they preferred in-person visits, while 8 (22.2%) respondents agreed or strongly agreed that they preferred telemedicine, and another 8 (22.2%) were neutral. In survey 2, these preferences were unchanged, with a slight shift in responses toward more favorable appraisals of telemedicine. However, a plurality continued to indicate a preference for in-person visits (disagree or strongly disagree: n=11, 45.8%). One-third (n=8, 33.3%) of respondents indicated a neutral response, with 20.8% (5/24) preferring telemedicine.

Overall, providers felt telemedicine was appropriate, with high proportions agreeing with statements that televisits provided the ability to communicate (survey 1: 29/36, 80.6%), talk privately about sensitive issues (26/36, 72.2%), and provide good quality care (26/36, 72.2%).

Most providers found telemedicine feasible. In survey 1, providers seemed to agree telemedicine saved them time (survey 1: 25/36, 69.4%), with half (survey 1: 18/36, 50%) agreeing they helped them be more productive. Respondents also reported high levels of confidence conducting televisits, meeting their patient needs using telemedicine, and compatibility with their schedules and ease of use. Fewer participants agreed that telemedicine was more effective (8/36, 22.2% favorable) and efficient (18/36, 50%).

Respondents indicated broad agreement with most of the survey items intended to address maintenance of telemedicine. Most providers strongly agreed or agreed with statements indicating support for telemedicine, that telemedicine was supported by clinical leadership, that they are open to working on new ways of using telemedicine, and that staff supported and were able to use telemedicine. Most providers wanted further feedback on their use of telemedicine. Overall, the reaction was neutral that staff had received sufficient training and coaching on telemedicine in survey 1.

**Figure 2.**
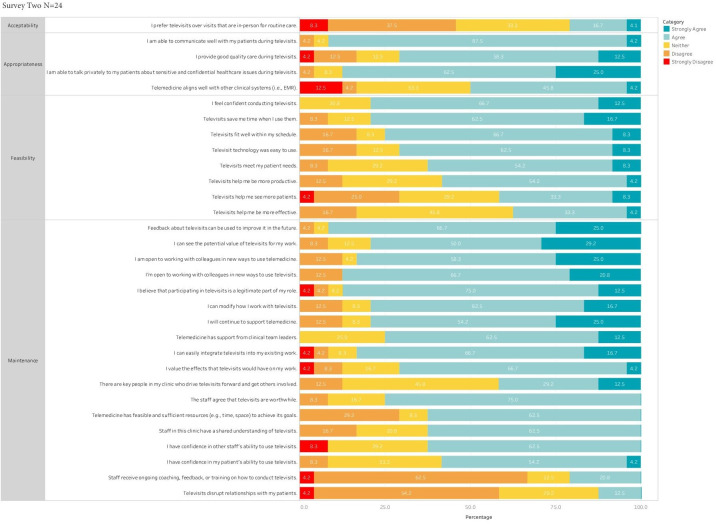
Survey 2.

Similar results around appropriateness, feasibility, and maintenance were seen in survey 2 (Figure 2). Questions assessing appropriateness showed overall high or higher proportions agreeing with the ability to communicate (survey 1: 29/36, 80.6% vs survey 2: 22/24, 91.7%), talk privately about sensitive issues (26/36, 72.2% vs 21/24, 87.5%), and provide good quality care (26/36, 72.2% vs 17/24, 70.8%). At survey 2, participants continued to increasingly agree that telemedicine saved them time (survey 1: 25/36, 69.4% and survey 2: 19/24, 79.2%) and helped them be more productive (survey 1: 18/36, 50% and survey 2: 14/24, 58.3%). Assessments of the effectiveness of televisits were more equivocal, although there was some evidence of a shift toward more favorable responses between survey 1 (8/36, 22.2%) and survey 2 (9/24, 37.5%). Notably, a lower proportion of respondents reported that telemedicine helped them see more patients in survey 2 (10/24, 41.7%) even after a long period of using the technology, compared to survey 1 (18/36, 50%). Regarding maintenance, there was overall concordance, except in 2 questions where even more providers disagreed with the statement that providers had received ongoing training and support (survey 1: 13/36, 36.1% agree and survey 2: 5/24, 20.8% agree). There seemed to be a decrease in reporting disruption in providers’ relationship with their patients from survey 1 (11/36, 30.6%) to survey 2 (3/24, 12.5%).

Better videoconferencing, access to laboratories, and software for sharing patient health care data, such as blood pressure, were recommended as ways to make telemedicine more valuable in the future.

### Implementation Outcomes With Differences by Site and Provider Type

Overall, there were few differences between sites in views regarding telemedicine (Table 2). More providers at HB indicated at least some potential value in telemedicine when compared to providers at NU (19/19, 100% at HB agree or strongly agree vs 14/17, 82.3% at NU; *P*=.04) in survey 1. In survey 2, all NU respondents indicated an ability to modify how they work with telemedicine (agree or strongly agree) versus 50% (5/10) of Howard Brown respondents (*P*=.002). No differences by site were found in both survey 1 and survey 2 among the items in the acceptability, appropriateness, and feasibility domains.

**Table 2.** Responses for individual Likert items demonstrating evidence of association by site or tenure.

Domain and item description		Response, n (%)					*P* value^a^	Total^b^
		Strongly agree	Agree	Neither agree nor disagree	Disagree	Strongly disagree		
Survey 1 by site								
Maintenance								
I can see the potential value of televisits for my work.							.04	
HB^c^ (n=22)		9 (47.4)	10 (52.6)	0	0	0		19
NU^d^ (n=21)		4 (23.5)	10 (58.8)	1 (5.9)	2 (11.8)	0		17
Total		13 (36.1)	20 (55.6)	1 (2.8)	2 (5.6)	0		36
Survey 1 by tenure								
Feasibility								
Televisits help me see more patients.							.008	
≤10 y (n=23)		3 (17.7)	9 (52.9)	5 (29.4)	0	0		17
>10 y (n=20)		1 (5.3)	5 (26.3)	7 (36.8)	6 (31.6)	0		19
Total		4 (11.1)	14 (38.9)	12 (33.3)	6 (16.7)	0		36
Appropriateness								
I am able to talk privately to my patients about sensitive and confidential healthcare issues during televisits.							.03	
≤10 y (n=23)		7 (41.2)	8 (47.1)	1 (5.9)	1 (5.9)	0		17
>10 y (n=20)		2 (10.5)	9 (47.4)	5 (26.3)	2 (10.5)	1 (5.3)		19
Total		9 (25.0)	17 (47.2)	6 (16.7)	3 (8.3)	1 (2.8)		36
Survey 2 by site								
Maintenance								
I can modify how I work with televisits.							.002	
HB (n=11)		0	5 (50)	2 (20)	3 (30)	0		10
NU (n=16)		4 (28.6)	10 (71.4)	0	0	0		14
Total		4 (16.7)	15 (62.5)	2 (8.3)	3 (12.5)	0		24
Survey 2 by tenure								
Appropriateness							.046	
Telemedicine aligns well with other clinical systems (ie, EMR)^e^.								
≤10 y (n=12)		0	2 (22.2)	4 (44.4)	1 (11.1)	2 (22.2)		9
>10 y (n=15)		1 (6.7)	9 (60)	4 (26.7)	0	1 (6.7)		15
Total		1 (4.2)	11 (45.8)	8 (33.3)	1 (4.2)	3 (12.5)		24
Acceptability								
I prefer televisits over visits that are in-person for routine care.							.02	
≤10 y (n=12)		1 (11.1)	3 (33.3)	3 (33.3)	2 (22.2)	0		9
>10 y (n=15)		0	1 (6.7)	5 (33.3)	7 (46.7)	2 (13.3)		15
Total		1 (4.2)	4 (16.7)	8 (33.3)	9 (37.5)	2 (8.3)		24

a *P* values calculated using exact Mantel-Haenszel methodologies. Missing values are not considered when calculating *P* values. Likert responses by site or tenure displayed for items with a *P* value <.05.

b Total refers to the number of nonmissing responses. This represents the denominator from which row percentages are calculated.

c HB: Howard Brown Health Center.

d NU: Northwestern University.

e EMR: electronic medical record.

There were some differences between providers based on tenure. In survey 1, providers with more than 10 years of experience were less likely to report that telemedicine helped them see more patients (31.6% agree or strongly agree vs 70.6% for providers with ≤10 years of experience; *P*=.008). Fewer experienced providers felt they could talk about private issues with their patients during telemedicine (57.9% of more experienced providers vs 88.3% of less experienced providers; *P*=.03). However, in survey 2, providers with more than 10 years of experience felt that visits were more integrated into their electronic medical record (66.7% agree or strongly agree vs 22.2% of less experienced providers; *P*=.046) but were less likely to prefer telemedicine to in-person care for routine care (6.7% agree or strongly agree vs 44.4% of less experienced providers; *P*=.02).

### Key Informant Interviews

A number of themes were found during the provider interviews that gave additional insights into their experience with telemedicine (Table 3).

**Table 3.** Important CFIR domains and constructs identified in key informant interviews.

Domains	Constructs	Determinants identified
Intervention characteristics	Adaptability Relative advantage	Perceptions regarding the benefits and barriers of telemedicine to continue their connection with patients
Inner setting	Tension for Change Compatibility Culture Recipient centeredness Structural characteristics Technology infrastructure	Need to consider the impact of the COVID-19 pandemic Staff have concerns over the availability of technology, patient engagement, and workflow disruption Staff from both sites lacked adequate and adapted spaces for conducting visits with adequate privacy
Outer setting	Patient needs or resources Cosmopolitanism	Patients experience stress, fear of the unknown, and disconnect with health care Staff feel a lack of support from local and governmental organizations on how to conduct telemedicine care, training on technologies, and support Linguistic barriers are exacerbated during telemedicine visits
Characteristics of individuals	Intervention knowledge or beliefs Other personal attributes Need	Knowledge and beliefs regarding technical aspects of telemedicine Need of the patients to receive care during the pandemic
Process	Tailoring strategies Engaging innovation recipients	Professionals adapted their visits, including additional support to attend to patients’ needs

### Theme 1: The Rapid Transition Due to COVID-19

The transition to telemedicine due to COVID-19 was rapid and unplanned, and providers saw themselves transitioning to a new reality of care in which their (physical) connection with their patients was severed, without considering disparities in the needs of those patients:

*Of course, there was a period of time where it really converted to pretty much all telemedicine. After we started seeing people in person, I would say I went quickly to half and half.*

*I have tried it on some of my patients who are really difficult to connect with [for example] people with substantial substance issues.*

Some providers adapted to televisits with ease, indicating very little change in their daily activities:

*I feel like it’s amazing how comfortable I feel doing telemedicine and feeling like I can provide as good care as when in person assuming that they’re relatively well and don’t have major issues* [...]

### Theme 2: Barriers to Telemedicine Visits

#### Overview

Although unplanned, most of the providers rapidly adjusted to this new configuration of care and communication, surprising even themselves with how quickly this adaptation process occurred. However, in situations where external (disruptions in the environment), media (the interface with technology), and internal (lack of patient engagement or focus) changes were occurring rapidly, providers reported barriers, including frustration on perceiving a disconnection with their patients and lacking other tools to overcome this at a distance.

#### Barrier: Lack of Support From the Institution

Facing these barriers, providers saw themselves without institutional support, having to adjust to this new reality without the proper tools to communicate and provide a quiet and private ambience to meet with their clients:

*[...] if they had the same level of attention to providing a space for us, I will not do a televisit with eight people in the room.*

#### Barrier: Privacy

Privacy was a main concern for providers. Providers could not replicate the same discretion of a clinic room while in telemedicine, having to resort to adjusting their speech to adverse situations, at the expense of clear communication. The main reason for this was the desire to protect their confidentiality at a time when many families were suddenly forced to remain confined in their houses, without a proper space to discuss sensitive subjects, like sexual health:

*There are times where they’re [the patients] like speaking in code. Because they’re at work and I’m trying to decipher what they’re trying to tell me it just depends on the situation [...], there’s other times when they’re speaking freely in front of like their grandma. You just never know what you’re going to get.*

#### Barrier: Accommodating Emotions During Telemedicine

Providers did not feel comfortable navigating more nuanced subjects during televisits with their patients, such as providing a diagnosis or being the bearer of “bad news” through a screen. Health professionals have developed skills to rely on body language and closer proximity to their patients to know how to handle emotional situations during in-person visits. When deprived of that in televisits, they felt unsure of how to conduct their care, physically and emotionally:

*Like interaction of catching people’s emotional responses or just like little nuances of being together in person it’s not the same in the video so I’m hearing like that human connection that kind of emotional cues are easier to find in in-person visits.*

*Well, telephone is more difficult because I feel like we end up interrupting each other in a way that we can’t see each other. So, I feel like that can impede both the communication but also just the comfort of the relationship.*

*I certainly don’t give people bad news that way if I have anticipated difficult news of course I try to bring them in on occasion I’ve had to give bad news over the phone I always ask the patient how they want it.*

#### Barrier: Difficulties in Language and Understanding

Providers expressed concerns about their ability to effectively manage and support patients when language and understanding were compromised. Providers found that telemedicine worked better with patients who could clearly understand and follow directions. For those who struggled with language, the absence of face-to-face interaction made it more difficult to ensure understanding and effective communication:

*I think it works better with a patient who is really capable and understands directions and that type of stuff.*

*Honestly I’m not real fond of using it for people who don’t speak English well I do use translators but it’s hard enough to use a translator in person but in person you’ve got the visual cues to help you too.*

### Theme 3: Opportunities and Adjustments

In contrast to the previous theme, demonstrating the diversity of experience collected from the providers, some found that telemedicine gave them the time and opportunity to finally connect to patients who had been more resistant to it before. This may be due to the alleviation of travel burden or more time available for the patients to attend visits.

Flexibility was a key point in the providers’ experiences, highlighted here when they mentioned that having the laboratories open during critical periods of the pandemic allowed them to continue their care with minimal disruption:

*There’s one guy who lives in downstate Illinois. I had one in-person visit with him, but I’ve been doing a lot of telemedicine because he really requires close follow-up. So, every month, I call him.*

*Or you end up having less time because you’ve taken more time for a physical exam or something that’s intervened, what you lack in a physical contact you make up for in in-depth of conversation something like that.*

Other aspects of the clinics being available during the lockdown revealed the benefits of flexibility to maintain certain functions:

*Luckily [the clinic] did keep its labs open, so we had the capacity to do labs on a semi-urgent basis.*

### Theme 4: Necessary Tools for Telemedicine

Providers stated that technology and communications tools were a central aspect of the telemedicine experience. Providers saw themselves being required to adapt quickly to incorporate those tools into their daily lives in a vital way. The implementation of televisits was conducted without necessarily providing them with specific tools, forcing them to adapt as situations presented themselves. For example, some providers used their personal phone numbers to maintain the best possible communication with their patients despite the risks that come with exposing personal information.

In many cases, providers reported creative solutions (eg, sharing their numbers, inviting a translator to a 3-way call, and sending pictures via text to take advantage of a high resolution) as a way of coping with a lack of institutional support tools dedicated to establishing a stable and safe communication channel with their patients, or proper training:

*[Talking about adaptations to telemedicine] is it a three-way call, [...] you call the translator first then they put the call into the patient they explain that they’re the translator and doctor is on the phone.*

*I actually have pretty much resorted to giving them my cell phone and asking them to send pictures because you don’t get a very good look at it [via zoom] that’s how I’ve augmented it.*

*[...] it’s something to think about or a way for a patient to send cell phone pictures confidentially to the provider without sending it to their cell phone there’s got to be a way to do that.*

While some participants seemed to be comfortable or even prefer using their phones, being a familiar technology, the use of personal devices by providers demonstrates a lack of institutional support. Instead of institutions providing technological support, the providers had to use their own devices to perform care:

*[...] it would be great if somebody actually showed me how to do the zoom call I actually finally grabbed “someone” and she’s like this is how you do it then we got it setup right.*

### Integration of Quantitative and Qualitative Findings (RE-AIM and CFIR)

To support a convergent mixed methods design, quantitative survey findings organized using the RE-AIM framework were integrated with qualitative interview findings mapped to the CFIR. Quantitative results describe implementation outcomes (eg, acceptability, adoption, and feasibility), while qualitative findings provide contextual explanations for these outcomes by identifying multilevel determinants influencing implementation. Table 4 presents a joint display aligning key RE-AIM outcomes with corresponding CFIR domains and representative qualitative insights.

**Table 4.** Integration of RE-AIM^a^ implementation outcomes with CFIR^b^-informed qualitative findings.

RE-AIM domain	Key quantitative finding (survey)	Relevant CFIR domains	Qualitative interpretation (KII^c^ themes)	Illustrative quote (example)
Reach	Most providers reported using telemedicine during the study period, with telephone visits used more frequently than video visits.	Outer setting: patient needs and resources	Providers described telemedicine (particularly telephone visits) as essential for reaching patients facing barriers such as limited internet access, unstable housing, or privacy concerns. However, limited visual assessment was noted as a trade-off.	“A lot of our patients just don’t have stable internet, so phone visits are really the only way to reach them.”
Adoption	Providers generally reported adopting telemedicine into their clinical practice, although uptake varied by role and clinical context.	Inner setting; characteristics of individuals	Adoption was influenced by provider comfort with technology, perceived fit with HIV care workflows, and prior experience. Some providers expressed greater enthusiasm than others.	“Some clinicians adapted quickly, others felt it didn’t fit how they normally deliver care.”
Acceptability	Telemedicine was rated as acceptable by most providers, particularly for follow-up and medication management visits.	Characteristics of individuals	Providers valued telemedicine for its flexibility and ability to maintain continuity of care but emphasized that acceptability decreased for visits requiring physical exams or sensitive discussions.	“It works well for routine follow-ups, but not everything can be done remotely.”
Implementation	Providers reported moderate feasibility of delivering care via telemedicine.	Intervention characteristics; process	While telemedicine was feasible, providers identified implementation challenges, including lack of standardized protocols, limited training, and uncertainty around documentation and billing procedures.	“We figured it out as we went along, but there wasn’t much guidance early on.”
Maintenance	Providers expressed uncertainty about the long-term sustainability of telemedicine beyond the COVID-19 pandemic.	Outer setting; inner setting	Concerns about future reimbursement policies, regulatory changes, and institutional support were prominent and influenced expectations about continued telemedicine use.	“If reimbursement changes again, it will really affect whether we can keep doing this.”

a RE-AIM: reach, effectiveness, adoption, implementation, and maintenance.

b CFIR: Consolidated Framework for Implementation Research.

c KII: key informant interview.

While overall quantitative data showed overall high appropriateness, feasibility, and maintenance, qualitative data highlighted concerns.

Concerns with privacy were more notable in KIIs than in the overall response to the survey questions, where most providers answered they were able to talk privately. Likewise, while most respondents agreed that they were able to communicate, qualitative responses showed many concerns around the ease of communication, especially for sensitive subjects, delivery of bad news, and for patients who do not speak English.

Both qualitative and quantitative data showed that feasibility and maintenance concerns correlated most with the inner setting domain around support for using the technology and proper staff and patient training. However, in the survey data, most respondents still agreed with questions that assessed if televisits were feasible and would be continued, while interviews often highlighted barriers they had faced. Possibly, the high degree of adaptability shown by the providers and some of the perceived benefits of the innovation identified in the KIIs ameliorated barriers, allowing for overall positive agreement with statements assessing feasibility.

Areas of agreement in the data showed that overall preference and acceptability for in-person visits remained higher with specific situations such as new patients, ill patients, and sensitive discussions given as examples in qualitative interviews.

The flexibility of telehealth in allowing patients to be seen who live far and providing more opportunity for discussion were also highlighted in provider interviews. As the majority of respondents in the survey agreed they felt they could adapt and work with leadership, staff, and patients to integrate and improve telehealth, the benefits reported could be strengthened and maintained.

## Discussion

### Principal Findings

In this mixed methods study conducted at 2 different health care settings in Chicago, most HIV providers reported that telemedicine visits were a feasible, acceptable, and appropriate method of delivering care during the pandemic and supported their continuation. While providers reported a relatively smooth transition to the emergent introduction of telemedicine in our surveys, in-depth interviews with providers identified significant confidentiality and privacy concerns and the potential for suboptimal communication in the virtual setting. These findings highlight the importance of monitoring telemedicine procedures while eliciting ongoing patient and provider input into how to best use this health care modality.

Overall, the uptake of telemedicine was high, although it did vary over the course of the study. Telemedicine was rapidly adopted and comprised a large proportion of visits initially in the COVID-19 pandemic, although it declined from one-half to one-third a year later (survey 2). This is similar to a recent review reporting up to 86.9% use of telemedicine at some HIV clinics after 2020 [18]. The overall reasons for maintaining telemedicine were not related directly to infection prevention but to factors around patient convenience and access, which may be driving use as the pandemic recedes. Furthermore, the importance of long-term engagement makes factors that improve patient access especially relevant to HIV care. Follow-up data on the use of telemedicine in HIV care and its effect on long-term patient engagement and adherence will be important.

During the initial phase of the COVID-19 pandemic, providers experienced a significant transition, moving swiftly from predominantly in-person visits to a mix of telemedicine and in-person consultations. Surprisingly, given this rapid expansion, HIV providers provided mostly positive responses in the surveys about telemedicine, reporting high levels of confidence with this mode of health care delivery. However, exploring this transition in more depth with providers during their interviews revealed important concerns about the impact it had on patient care. At least one provider reported that the transition occurred without adequate consideration for the varying needs and circumstances of patients, while others were concerned about an apparent disconnect with patients. Despite these concerns, there was significant support for continuing to support telemedicine in the future, as indicated in individual responses in the maintenance domain of the provider survey.

Although most providers in the survey indicated that telemedicine was appropriate in that they could communicate and speak privately with patients, interviews indicated that privacy and confidentiality were frequent concerns. Confidentiality and privacy concerns have been shown to be a major barrier to accessing HIV care. In a survey of 80 people living with HIV and 60 HIV physicians in Italy, physicians had more concerns than people living with HIV that telemedicine use would increase isolation, create difficulties in interaction, worsen the disconnect between patients and providers, and result in a lower quality of care for patients. Both groups agreed they were willing to use telemedicine but that it should not replace personal visits completely [34]. Another recent study in Florida, United States, found that some people living with HIV felt they faced more potential confidentiality concerns with in-person care, especially if they received care in a clinic solely focused on HIV, but others had more privacy concerns with telehealth [35]. In our study, privacy was less of a concern in survey 2, possibly as clinics had more consistent procedures in place for delivering telemedicine and patients became more comfortable participating in televisits in their local environments. Other work has found that providers noted the ability to assess their patients’ home environments could actually be a benefit [36], but this was not commonly noted in our study.

Adequate communication is a major component of appropriate health care and was a common theme identified by providers. Moreover, trust in health care workers requires communication with respect, partnership, and appropriate time [37]. Trust in their providers has been linked to better health literacy, better retention in care, and antiretroviral therapy adherence for people living with HIV [38]. Overall, providers responded that they could communicate well with their patients in the surveys; however, during interviews, they expressed concerns about the lack of nonverbal cues and physical contact to deliver bad news and discuss emotional issues. Providers also noted that many visits require a physical examination to be effective and expressed concern about a loss of connection, including lack of physical touch, which is important to the provider-patient relationship [36]. Literature suggests patients overall do not prioritize physical connection nor the need for physical diagnosis as much as providers [39]. Our study suggests that telemedicine may not fully replicate the interpersonal dynamics and communication of in-person consultations, particularly in emotionally charged situations and with vulnerable populations.

Some providers did note that telemedicine visits could provide more time for discussion compared to in-person visits, thereby improving access due to more flexibility on both the patient and provider side and the decreased need for travel. A similar observation was made by Walker et al [40] in assessing provider attitudes to telemedicine in California, United States. In this study, providers felt telemedicine reduced patient barriers to care and offered increased flexibility to providers, although there were concerns over the complexity of workflows for telemedicine, technical challenges with video platforms, and an overall high degree of variation among provider views. In a separate qualitative study of HIV providers, there was overall support for patients and staff to continue telemedicine, with perceptions that telemedicine was useful to keep patients in care, reporting specifically that it was easier and more flexible for patients. However, the authors also cited technology challenges, such as a lack of smartphones for both patients and providers in addition to reports of poor connectivity during visits and privacy concerns [41].

In our study, we found most visits did not use video, which perhaps reflected uncertainty or hesitancy using this then-new technology. Similar observations have been made in other studies [3,40]. A recent systematic review showed video may have better diagnostic accuracy than phone for acute conditions or those requiring physical findings, but in many outpatient settings, clinical outcomes were equivalent for phone or video visits [42]. Interestingly, patient satisfaction was noninferior for telephone compared to video televisits in 1 study [43]. Given the rapid rollout of telemedicine during this study, it is unclear if increasing video use would have increased provider frustration or positively or negatively affected their privacy concerns. Further information on the acceptability and efficacy of telephone versus video modalities is needed, especially for HIV care, along with clarity regarding reimbursement for the differing types of visits.

Despite the challenges, the study identified several opportunities associated with telemedicine. Some providers reported improved connectivity with patients who had previously been resistant to traditional health care settings. Flexibility emerged as a key advantage, allowing for increased access to care and more efficient use of time for both providers and patients. The availability of essential health care services, such as laboratory testing, during lockdowns underscored the resilience of telemedicine in maintaining continuity of care during crises.

### Limitations

The strengths of our study include a heterogeneous sample of providers of different backgrounds and experience at 2 different sites (academic and community health center) and 2 different periods. However, our study suffered from an inability to track a given provider’s responses across survey administrations and small sample sizes that, taken together, limited our ability to apply statistical inferences. Changes in attitudes over time for participants were not considered, as respondents’ identifiers were randomly assigned between survey administrations and unlinked to participants’ personal identifiers to further maintain anonymity from the prospective peer respondents, which prevented the possibility of paired analyses. We did generate mean domain scores that were intended to summarize feasibility, maintenance, and appropriateness, with each constituent Likert item scored on a scale of 1 to 5, with higher scores indicating a more favorable assessment. These summaries can be found in Table S2 in Multimedia Appendix 2 (available on request). Summarizing consolidated items by domain revealed largely equivocal results. Although there is some evidence of a difference in the assessment of appropriateness by tenure in the first survey, we had some concerns regarding the raw Cronbach α of 0.672 obtained when testing this domain using these data. Given these findings and a primary interest in item-specific differences by site and tenure, we opted to exclude these analyses. We also considered and tested the possibility of a multivariable analysis of these data using proportional odds models, where the 5-category Likert outcomes were consolidated into 3 categories (ie, agree, neither agree nor disagree, and disagree). Sample sizes made this difficult, however, and the interpretation of the resulting odds ratios was a concern. Ultimately, we determined that reporting a simple analysis emphasizing the descriptive results with a minimum of applied inference was a better approach. In addition, qualitative and quantitative responses from individual participants could not be compared.

The single urban setting from only 2 clinics limited the generalization of our results to other settings. It should be noted that a relatively high rate of providers in both clinics chose not to respond to the survey, which possibly reflected the demands of COVID-19 on providers at that time. Therefore, results may reflect a response bias toward those with either more positive or more negative views. Additional limitations include reliance on self-reported attitudes rather than objective provider metrics, such as visit number and duration, and a potential that responses may suffer from social desirability bias as participants were employees of the organization. Nonetheless, this study demonstrates an initial indication that characterizes the use of telemedicine for HIV care as largely acceptable, feasible, appropriate, and sustainable. These findings recommend the implementation of larger, robust longitudinal investigations rigorously evaluating a potentially promising means of augmenting and facilitating HIV patient care.

### Conclusions

In this mixed methods study conducted at 2 different health care settings in Chicago, most HIV providers reported that telemedicine visits overall were a feasible, acceptable, and appropriate method of delivering care during the pandemic and supported their continuation. The findings of this study have several implications for future health care practice and policy. Addressing the identified challenges, such as privacy concerns and the need for institutional support, will be crucial in ensuring the sustainability and effectiveness of telemedicine initiatives. Investments in technological infrastructure, training programs, and regulatory frameworks are essential to maximize the potential of telemedicine while mitigating its limitations. Additionally, future research should focus on longitudinal studies to assess the long-term impacts of telemedicine on patient outcomes and health care delivery models, especially in HIV care. Overall, our results suggest that support for telemedicine as a continuing care modality is warranted.

## Supplementary material

10.2196/75933 Multimedia Appendix 1 Survey questions and domains.

10.2196/75933 Multimedia Appendix 2 Supplementary table.

## Abbreviations

CFIR: Consolidated Framework for Implementation Research
HB: Howard Brown Health Center
HIPAA: Health Insurance Portability and Accountability Act
KII: key informant interview
NU: Northwestern University Infectious Diseases Clinic
RE-AIM: reach, effectiveness, adoption, implementation, and maintenance
REDCap: Research Electronic Data Capture
