# Supplementary material for: Rapid Deployment of Telemedicine in HIV Care: Mixed Methods Study of Providers’ Attitudes and Perceptions
Source: J Med Internet Res. 2026 Apr 27;28:e75933. doi: 10.2196/75933 (PMC13120543; doi:10.2196/75933)
Supplement: Multimedia Appendix 1 [file jmir-v28-e75933-s001.docx]

Supplemental Table 1: Survey Questions and Domains

| Acceptability | I prefer televisits over visits that are in-person for routine care. |
| --- | --- |
| Appropriateness | Telemedicine aligns well with other clinical systems (i.e. EMR). |
| Appropriateness | I am able to talk privately to my patients about sensitive and confidential  healthcare issues during televisits. |
| Appropriateness | I provide good quality care during televisits. |
| Appropriateness | I am able to communicate well with my patient during televisits. |
| Feasibility | Televisits help me be more effective. |
| Feasibility | Televisits help me to be more productive. |
| Feasibility | Televisits save me time when I use them. |
| Feasibility | Televisits meet my patient needs. |
| Feasibility | I feel confident conducting televisits. |
| Feasibility | Televisit technology was easy to use. |
| Feasibility | Televisits fit well within my schedule. |
| Feasibility | Televisits help me see more patients. |
| Maintenance | Telemedicine has support from clinical team leaders. |
| Maintenance | Staff receive ongoing coaching, feedback, or training on how to conduct telemedicine |
| Maintenance | Telemedicine has feasible and sufficient resources (e.g., time, space) to achieve its goals |
| Maintenance | I am open to working with colleagues in new ways to use telemedicine |
| Maintenance | I will continue to support telemedicine. |
| Maintenance | Staff in this clinic have a shared understanding of televisits. |
| Maintenance | I can see the potential value of televisits for my work. |
| Maintenance | There are key people in my clinic who drive televisits forward and get others involved. |
| Maintenance | I believe that participating in telemedicine is a legitimate part of my role. |
| Maintenance | I'm open to working with colleagues in new ways to use televisits. |
| Maintenance | I can easily integrate televisits into my existing work. |
| Maintenance | Televisits disrupt relationships with my patients. |
| Maintenance | I have confidence in other staff's ability to use televisits. |
| Maintenance | I have confidence in my patient's ability to use televisits. |
| Maintenance | The staff agree that televisits are worthwhile. |
| Maintenance | I value the effects that televisits would have on my work. |
| Maintenance | Feedback about televisits can be used to improve it in the future. |
| Maintenance | I can modify how I work with televisits. |
