# Supplementary material for: Rapid Deployment of Telemedicine in HIV Care: Mixed Methods Study of Providers’ Attitudes and Perceptions
Source: J Med Internet Res. 2026 Apr 27;28:e75933. doi: 10.2196/75933 (PMC13120543; doi:10.2196/75933)
Supplement: Multimedia Appendix 2 [file jmir-v28-e75933-s002.docx]

|  | **Feasibility Score** | | | | **Maintenance Score** | | | | **Appropriateness Score** | | | |
| --- | --- | --- | --- | --- | --- | --- | --- | --- | --- | --- | --- | --- |
| **By Site** |  |  |  |  |  |  |  |  |  |  |  |  |
|  | **HB** | **NU** | ***P*-val** | **Total** | **HB** | **NU** | ***P*-val** | **Total** | **HB** | **NU** | ***P*-val** | **Total** |
| First Survey Administration |  |  |  |  |  |  |  |  |  |  |  |  |
| n | 19 | 17 |  | 36 | 19 | 17 |  | 36 | 19 | 17 |  | 36 |
| Mean (SD) | 2.37 (0.740) | 2.60 (0.627) |  | 2.48 (0.689) | 2.15 (0.455) | 2.28 (0.537) |  | 2.22 (0.492) | 2.20 (0.675) | 2.29 (0.741) |  | 2.24 (0.698) |
| Median | 2.38 | 2.50 | .50 | 2.44 | 2.11 | 2.22 | .86 | 2.14 | 2.25 | 2.00 | .87 | 2.13 |
| Min, Max | 1.0, 3.6 | 1.8, 3.6 |  | 1.0, 3.6 | 1.2, 2.9 | 1.7, 3.7 |  | 1.2, 3.7 | 1.3, 3.5 | 1.3, 4.0 |  | 1.3, 4.0 |
|  |  |  |  |  |  |  |  |  |  |  |  |  |
| Second Survey Administration |  |  |  |  |  |  |  |  |  |  |  |  |
| n | 10 | 14 |  | 24 | 10 | 14 |  | 24 | 10 | 14 |  | 24 |
| Mean (SD) | 2.38 (0.520) | 2.46 (0.560) |  | 2.42 (0.534) | 2.35 (0.224) | 2.33 (0.656) |  | 2.34 (0.512) | 2.35 (0.516) | 2.23 (0.631) |  | 2.28 (0.577) |
| Median | 2.31 | 2.25 | .86 | 2.31 | 2.31 | 2.25 | .43 | 2.25 | 2.25 | 2.13 | .47 | 2.25 |
| Min, Max | 1.6, 3.4 | 1.8, 3.6 |  | 1.6, 3.6 | 2.1, 2.8 | 1.6, 3.7 |  | 1.6, 3.8 | 1.8, 3.3 | 1.5, 4.3 |  | 1.5, 4.3 |
|  |  |  |  |  |  |  |  |  |  |  |  |  |
| **By Tenure** |  |  |  |  |  |  |  |  |  |  |  |  |
|  | **<= 10 years** | **> 10 years** |  |  | **<= 10 years** | **> 10 years** |  |  | **<= 10 years** | **> 10 years** |  |  |
|  |  |  |  |  |  |  |  |  |  |  |  |  |
| First Survey Administration |  |  |  |  |  |  |  |  |  |  |  |  |
| n | 17 | 19 |  | 36 | 17 | 19 |  | 36 | 17 | 19 |  | 36 |
| Mean (SD) | 2.29 (0.666) | 2.64 (0.686) |  | 2.48 (0.689) | 2.13 (0.429) | 2.29 (0.542) |  | 2.22 (0.492) | 2.03 (0.723) | 2.43 (0.634) |  | 2.24 (0.698) |
| Median | 2.25 | 2.50 | .12 | 2.44 | 2.00 | 2.22 | .38 | 2.14 | 2.00 | 2.25 | .01 | 2.13 |
| Min, Max | 1.0, 3.6 | 1.0, 3.6 |  | 1.0, 3.6 | 1.2, 3.1 | 1.3, 3.7 |  | 1.2, 3.7 | 1.3, 3.5 | 1.3, 4.0 |  | 1.3, 4.0 |
|  |  |  |  |  |  |  |  |  |  |  |  |  |
| Second Survey Administration |  |  |  |  |  |  |  |  |  |  |  |  |
| n | 9 | 15 |  | 24 | 9 | 15 |  | 24 | 9 | 15 |  | 24 |
| Mean (SD) | 2.21 (0.353) | 2.55 (0.592) |  | 2.42 (0.534) | 2.19 (0.282) | 2.43 (0.603) |  | 2.34 (0.512) | 2.28 (0.423) | 2.28 (0.667) |  | 2.28 (0.577) |
| Median | 2.25 | 2.38 | .32 | 2.31 | 2.22 | 2.28 | .40 | 2.25 | 2.25 | 2.00 | .60 | 2.25 |
| Min, Max | 1.6, 2.8 | 1.9, 3.6 |  | 1.6, 3.6 | 1.7, 2.5 | 1.6, 3.8 |  | 1.6, 3.8 | 1.8, 3.0 | 1.5, 4.3 |  | 1.5, 4.3 |
| *P*-values calculated using two-sided Wilcoxon Rank Sum tests employing an exact calculation. In cases of ties, average scores or midpoint ranks were used. | | | | | | | | | | | | |
